# Associations between alcohol use and peripheral, genetic, and epigenetic markers of oxytocin in a general sample of young and older adults

**DOI:** 10.1002/brb3.2425

**Published:** 2022-02-11

**Authors:** Jillian M. Rung, Quintin A. Kidder, Marilyn Horta, H. P. Nazarloo, C. Sue Carter, Meredith S. Berry, Natalie C. Ebner

**Affiliations:** ^1^ Department of Psychology University of Florida Gainesville Florida USA; ^2^ Department of Epidemiology University of Florida Gainesville Florida USA; ^3^ Department of Health Education and Behavior University of Florida Gainesville Florida USA; ^4^ Kinsey Institute Indiana University Bloomington Indiana USA; ^5^ Department of Psychology University of Virginia Charlottesville Virginia USA; ^6^ Pain Research and Intervention Center of Excellence (PRICE) College of Medicine Clinical and Translational Science Institute (CTSI) University of Florida Gainesville Florida USA; ^7^ Department of Aging & Geriatric Research Institute on Aging University of Florida Gainesville Florida USA

**Keywords:** alcohol, DNA methylation, genetic predisposition, genotype, oxytocin, peripheral, polymorphism, substance use

## Abstract

**Introduction:**

Human and nonhuman animal research suggests that greater oxytocin (OT) activity is protective against harmful substance use. Most research on this topic is preclinical, with few studies evaluating the association between substance use and individual differences in the human OT system. The present study sought to fill this gap by evaluating the relationship between alcohol use and multiple biological measures of OT activity in an overall low to moderate‐drinking sample.

**Method:**

As part of a larger study, generally healthy young (*n* = 51) and older (*n* = 53) adults self‐reported whether they regularly used alcohol and how much alcohol they consumed per week. Participants also provided blood samples from which peripheral OT, and in an age‐heterogeneous subset of participants (*n* = 56) variation in the oxytocin receptor gene (the *OXTR* rs53576 polymorphism) and *OXTR* DNA methylation levels (at cytosine–guanine dinucleotide sites ‐860, ‐924, ‐934), were obtained.

**Results:**

A‐allele carriers of the *OXTR* rs53579 polymorphism were less likely to regularly consume alcohol. Among regular alcohol consumers, number of alcoholic drinks per week was positively associated with peripheral OT in regression models excluding observations of high influence (postdiagnostic models). Number of alcoholic drinks per week was consistently negatively associated with *OXTR* DNA methylation at site ‐860; and with *OXTR* DNA methylation at site ‐924 in postdiagnostic models.

**Conclusions:**

The significant associations between alcohol use and individual differences in OT activity support the involvement of the OT system in alcohol use, which most likely reflect the role of OT when alcohol use is under control of its rewarding properties and/or the acute impacts of alcohol on the OT system. Additional research with markers of OT activity and alcohol use, particularly longitudinal, is needed to clarify the bidirectional effects of OT and alcohol use in moderate to harmful drinking and dependence.

## Graphical abstract

Multiple measures thought to be indicative of lower oxytocin activity were associated with less frequent (i.e., non‐regular consumption) and fewer alcoholic beverages consumed per week in younger and older adult humans engaging in generally low to moderate alcohol use. These associations were most robust between regular alcohol use and variation in the oxytocin receptor gene (the rs53576 polymorphism); and degree of oxytocin receptor gene DNA methylation at specific sites and quantities of alcohol consumed.

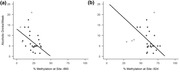

## 1 INTRODUCTION

Oxytocin (OT) is a nine‐amino neuropeptide involved in a wide range of social and nonsocial processes and behaviors that are crucial for survival (Quintana & Guastella, 2020), including reproduction (Gimpl & Fahrenholz, 2001), food intake (Lawson, 2017), stress response (Matsushita et al., 2019), and pain regulation (Rash & Campbell, 2014). Largely preclinical work has established that endogenous OT activity is regulated by the oxytocin receptor gene (*OXTR*; Jurek & Neumann, 2018) with notable impacts on social behavior (Carter, 2017; Donaldson & Young, 2008), including the perception of social signals (Marlin et al., 2015), transmission of maternal behaviors (e.g., alloparenting; Carcea et al., 2021), as well as the facilitation of social memory (Ferguson et al., 2000) and pair‐bonding (Carter & Perkeybile, 2018; Young & Wang, 2004).

The endogenous OT system also plays a role in modulating substance use behaviors as well as the etiological development of substance use dependence, though the mechanisms in these contexts are not yet well understood. For example, individual differences in the OT system (e.g., genetic variation) may confer susceptibility to substance use and substance use disorders through direct and indirect influences on the effects of drugs as well as through interactions with neurobiological (e.g., stress, reward) and immune systems (Buisman‐Pijlman et al., 2014). Further, findings on exogenous OT‐related alterations in tolerance, withdrawal, sensitization, and substance seeking/intake behaviors (King et al., 2020) have supported the potential use of OT administration for substance use disorder treatment (Horta et al., 2020; Lee et al., 2016; Peris et al., 2020).

Conceptual evidence for the role of OT in substance use primarily lies in shared factors that modify the OT system and its activity and substance use. As with other brain systems, early life experience appears critical in shaping the OT system (Buisman‐Pijlman et al., 2014; Ellis et al., 2021). Preclinical studies demonstrate positive associations between levels of maternal care and plasma OT in rats (Henriques et al., 2014), and between peer interactions in rat pups with *OXTR* levels and affiliative behavior in adulthood (Branchi et al., 2013). Preclinical models involving lower levels of care and social interaction are considered parallels to early life adversity and poorer social connection/support in humans. Additionally, different types of early life adversity (i.e., child abuse, early life stress) have been negatively associated with plasma OT levels in both women (Heim et al., 2009) and men (Opacka‐Juffry & Mohiyeddini, 2012). These changes in the OT system in response to adverse environments and experiences may be considered as an adaptive, preparatory response that imparts certain advantages (e.g., Ellis et al., 2021), but such benefits may be limited to certain contexts and/or confer risks in others (Ellis et al., 2021; Quintana & Guastella, 2020). That early adversity is a significant predictor of substance use in humans (De Bellis, 2002)—as well as for some types of substance use in adult nonhumans (see Baracz et al., 2020, for a review by drug and developmental stages)—illustrates the ways that such adaptations may confer contextually dependent benefits.

Variation in the rs53576 single nucleotide polymorphism (SNP) of the *OXTR* gene and methylation of *OXTR* promoter regions also appear to capture meaningful individual differences in the OT system. The *OXTR* gene is responsible for encoding OT receptors (Kimura et al., 1992) that mediate OT functions (Breton & Zingg, 1997); and greater methylation of *OXTR* promoter regions is associated with reduced transcription and expression of *OXTR* (Gregory et al., 2009; Kusui et al., 2001). Homo‐ and heterozygous A‐allele carriers of the *OXTR* rs53576 are thought to have less efficient OT signaling (Bakermans‐Kranenburg & van Ijzendoorn, 2008; Marsh et al., 2012), and greater methylation of *OXTR* is associated with reduced functional connectivity of brain regions involved in social cognition in healthy individuals (Puglia et al., 2018, 2015). *OXTR* methylation appears implicated in psychiatric conditions and stress, with higher and lower levels associated with obsessive‐compulsive disorder and anxiety disorders, respectively; and lower levels of *OXTR* methylation in infants were associated with greater levels of maternal stress (see Maud et al., 2018, and Kraaijenvanger et al., 2019, for reviews). In contrast, acute stress in adulthood increases *OXTR* methylation (Unternaehrer et al., 2012).

Despite the variety of measures that capture individual differences in the OT system and its activity, there are only a few studies directly evaluating their association with substance use in humans. A longitudinal study in young men found that individuals with the A/A genotype of the *OXTR* rs53576 SNP engaged in more frequent alcohol use and were more likely to demonstrate alcohol misuse or dependence by age 25 (Vaht et al., 2016). In a retrospective study, among males who completed suicide and had detectable levels of blood alcohol, homozygous A‐allele carriers had higher blood alcohol concentrations (Wasilewska et al., 2017). In this same report, however, there was no significant genotypic association with daily alcohol consumption in a separate, general population sample. More recently, *OXTR* methylation has been found to both directly and indirectly (i.e., as a mediator) predict future substance use–related problems (e.g., social or other difficulties resulting from alcohol or other drug use) in a sample of young African American men (19–22 years old) recruited from impoverished locales (Kogan et al., 2018). Together, biological measures indicating lower OT activity may serve as an important biomarker for substance use disorder risk; but the utility of some measures of OT activity (i.e., plasma OT) and their relevance to substance use across levels of use is unclear (i.e., more casual use under control of alcohol reward vs. heavier use that has transitioned to dependence).

In light of the lack of research on this topic, the goal of the present study was to provide a concurrent, more comprehensive examination of the association between individual differences in OT activity and substance use in humans. In a sample of young and older adults who generally consumed alcohol at low to moderate levels, we sought to evaluate the relationship between alcohol use and endogenous levels of plasma OT as well as genetic and epigenetic regulation in OT activity. We hypothesized that (i) higher levels of alcohol consumption would be associated with lower peripheral levels of plasma OT; (ii) regular alcohol use would be associated with *OXTR* polymorphisms previously linked to problematic alcohol use (e.g., A‐allele carriers of the *OXTR* rs53576); and (iii) greater alcohol use would be associated with epigenetic modification of OT function (i.e., greater DNA methylation of *OXTR* promoter regions).

This research serves as an important step in lending empirical support to existing theories of the role of individual differences in the OT system and OT activity in human substance use, and specifically prior to the onset of a use disorder (Buisman‐Pijlman et al., 2014). It furthermore extends prior work on this topic to include an age‐heterogeneous sample (i.e., young adults aged 18 to 31 and older adults aged 63 to 80 years); and it encompasses the first evaluation, to our knowledge, of the relationship between continuous measures indicative of individual differences in the OT system (i.e., degree of *OXTR* methylation) and substance use itself (cf. Kogan et al., 2018, who evaluated substance‐related problems, but not quantities consumed).

## 2 MATERIAL AND METHODS

### 2.1 Sample

The present data (*N *= 105) were collected as part of a larger study on the effects of a single dose (24 IUs) of intranasal OT on different facets of socioemotional functioning (e.g., social decision making and judgments of others; clinicaltrials.gov, NCT01823146). Results for the primary aims and other research questions are reported elsewhere (e.g., Ebner et al., 2016, 2015, 2019; Frazier et al., 2021; Horta et al., 2019; Plasencia et al., 2019). Participants were recruited through multiple sources: HealthStreet, which is a university‐affiliated community engagement program that helps identify potentially eligible individuals for research; IRB‐approved participant registries; flyers as well as handouts distributed in communities in Alachua County, FL, and word‐of‐mouth.

Individuals who expressed interest in participating in the study were provided a thorough explanation of study procedures and completed a prescreening assessment over the phone to determine initial eligibility. In this prescreening, researchers confirmed the participant's age was within one of two targeted ranges: between 18 and 30 years (young) or between 63 and 85 years (older). One exception was made to these criteria for one young adult who initially met age criteria at screening and turned 31 between the screening procedures and enrollment in the primary study. Only individuals identifying as White were eligible to participate in this first study on this topic due to previous reports indicating that frequencies of *OXTR* genotypes vary with race and/or ethnicity as does their association with certain conditions (e.g., autism spectrum disorder; Liu et al., 2010). Subsequently, the Telephone Interview for Cognitive Status (TICS; Brandt et al., 1988) was administered to older adults. If older participants scored 30 or higher (cognitively unimpaired) they were eligible for continuation of the screening process. Both young and older adults then responded to the MRI Eligibility questionnaire (Ebner et al., 2016; Frazier et al., 2021; Horta et al., 2019). The final portion of the phone screening consisted of health and demographics questions, with the former being used to identify participants who met the remaining inclusion criteria: being in generally good health, not pregnant or breastfeeding, no significant cognitive deficits, and no recent major surgeries or operations (see Horta et al., 2019, for a complete list of criteria).

A total of 105 young (24 female, 27 male) and older (30 female, 24 male) adults qualified for inclusion and participated in the larger study. Most young women were in the luteal phase of their menstrual cycle (22 of 24, or 92%). Two older adults were on hormone replacement therapy: one female and one male. The latter participant did not provide a blood sample and was excluded from analyses because these measures were key to the aims of the present report. Within this sample of 104, a subset of 56 participants had genome sequencing completed. Details on sample collection and derivation of measures from biological samples are described in the procedures, in Section 2.2.3.

### 2.2 Procedures

Participants who were initially deemed eligible based on their responses in the phone prescreening were invited to complete a more thorough screening assessment in the laboratory. This in‐person screening visit took place between 2 and 10 days after completing the phone prescreening. At the laboratory screening, the study procedures were described in full and research staff obtained participants’ written informed consent. Next, participants completed a more detailed health review and cognitive/socioemotional measures (not reported/analyzed herein; see Ebner et al., 2019; Lin et al., 2018; and Plasencia et al., 2019), after which blood samples were obtained (see Section 2.2.3). Participants were compensated upon conclusion of the screening visit. Measures from the primary study sessions are not included in the present report and thus these sessions are not discussed (see, e.g., Ebner et al., 2015; Horta et al., 2019; Frazier et al., 2021, for details). All study procedures were approved by the University of Florida Institutional Review Board.

#### 2.2.1 Demographics and basic health information

Demographic and basic health information needed to determine initial eligibility and characterize the sample was collected during the phone prescreening. This information included race, ethnicity, years of education, self‐rated physical and mental health, and current regular and past alcohol use (see Table S1). Physical and mental health were rated on a 1–10 scale, with 1 being poor and 10 being excellent.

#### 2.2.2 Alcohol use measures

Alcohol use was obtained during the phone prescreening. Participants were first asked if they consumed alcohol regularly. If they reported regular use, participants were then asked how much alcohol (i.e., number of drinks) they consumed per week using an open‐ended response format. After reporting on current use, participants were asked several questions about past alcohol use, but due to inconsistencies in the manner that responses were provided, these data are not analyzed herein. The specific questions and corresponding data are provided on the Open Science Framework (Rung et al., 2020). Participants were asked these same questions for caffeine, nicotine, and recreational drug use, but due to the infrequent reporting of the latter types of substance use these data are not included in the analyses here.

#### 2.2.3 Blood collection and biological measures

Blood samples (plasma and whole blood) were collected from participants to measure peripheral plasma OT, and in a subset, determine *OXTR* genotypes and *OXTR* DNA methylation levels. Samples were centrifuged and stored according to testing/manufacturer guidelines. Plasma OT was assayed without extraction using an Enzyme Immunoassay (EIA) purchased from Enzo LifeSciences, Inc. (Farmingdale, New York). While unextracted samples yield higher concentrations than extracted samples, unextracted samples provide accurate assessments of OT in human blood plasma (Carter et al., 2007; MacLean et al., 2019) that are more likely to be correlated with measures of behavior; this has been found in our laboratory (Roels et al., 2021) and in those of other scientists (Chu et al., 2020; Saxbe et al., 2019). For further details on the decision to not use extraction see Ebner et al. (2019) and Plasencia et al. (2019). *OXTR* DNA methylation levels were assessed at three cytosine–guanine dinucleotide (CpG) sites (−860, −924, −934). The sites were chosen based on those that evidenced significantly greater levels of methylation in individuals with autism (Gregory et al., 2009), which was one of the few existing publications on *OXTR* methylation at the time of assay. Full details on blood collection procedures as well as the assessment of plasma OT and *OXTR* DNA methylation levels are provided in Ebner et al. (2019) and Plasencia et al. (2019). Correlations between plasma OT and *OXTR* methylation measures are shown in Table S2. Genotyping for *OXTR* rs53576 was completed with whole blood samples using pyrosequencing. A/A and G/A genotype carriers were collapsed due to low frequency of A/A genotypes, as in prior research (e.g., Kumsta & Heinrichs, 2013). The combined A‐allele carriers are subsequently referred to as X/A.

(Epi)genotyping was conducted on only a subset of participants. This was because the idea and opportunity to conduct these assays arose after data collection had already started. There were no significant differences in sample descriptive measures (e.g., years of education, self‐rated physical and mental health) or plasma OT levels between those individuals whose samples were (epi)genotyped and those whose samples were not. However, instituting this assay later in recruitment resulted in a significantly higher proportion of older adults (.60) in the (epi)genotyped sample (vs. .41, *p* = .048, *V* = .21). Details of these comparisons are not reported in the text but can be generated from the analysis code provided on the Open Science Framework (Rung et al., 2020).

### 2.3 Statistical analysis

Prior to analysis, alcohol use data were recoded. When these data were collected, research assistants recorded the data in varying formats (e.g., a range of alcoholic drinks per week, in bottles vs. glasses of wine). Thus, recoding data consisted of standardizing responses into a single number and unit. Recoding procedures are detailed in documentation provided on the Open Science Framework (Rung et al., 2020).

Prior to conducting statistical tests addressing the primary aims in this paper, differences in demographics, alcohol use, genotype frequencies, and DNA methylation between the four targeted recruitment groups (young men, young women, older men, older women) were assessed using ANOVAs (or Kruskall‐Wallis tests) and Fisher's exact tests for continuous and categorical variables, respectively. These tests were conducted to ensure the targeted recruitment groups did not introduce potential confounds in tests of the primary hypotheses, which were planned to be evaluated without regard to these groupings. For continuous variables, ANOVAs were used when distributions of the variables within each of the four groups met assumptions of normality using Shapiro–Wilks tests. No significant differences were found across groupings for any of the measures (see Tables S1, S3, and S4) and thus are not further discussed.

Statistical analysis for the aims of interest was as follows. First, the relation between alcohol use and endogenous plasma OT was assessed using bivariate regressions with plasma OT predicting the number of alcoholic drinks per week. Second, the relation between *OXTR* rs53576 polymorphisms and alcohol use was assessed using Fisher's exact and Wilcoxon rank‐sum tests. In other words, we tested whether the number of participants endorsing regular alcohol use and the number of alcoholic drinks per week significantly differed as a function of *OXTR* rs53576 genotype. Finally, the relationship between levels of *OXTR* methylation at the −860, −924, and −934 sites and alcohol use was evaluated using separate bivariate regressions, one for methylation measures at each of the CpG sites.

Models were conducted using all observations, from which both Cook's distance (*D*) and leverage (*h*) were calculated. Observations that were high influence (*D* ≥ 4/*n*) or high leverage (*h* ≥ $2\times [\frac{k+1}{n}]$; where *k* is the number of predictors and *n* is the number of observations) were flagged and then excluded from the model. Both the results of analyses with (i.e., all observations) and without these influential/high leverage values (i.e., postdiagnostics) are reported.

The statistical program R (R Core Team, 2018) was used for analyses. Effect sizes for Wilcoxon and Kruskal–Wallis tests are the estimated eta‐squared (η^2^) using the rstatix package (Kassambara, 2020); and effect sizes reported for Fisher's exact tests are bias‐corrected Cramer's *V* using the rcompanion package (Mangiafico, 2020). For η^2^, effect sizes of .01 to .06 are considered small, greater than .06 to .14 moderate, and greater than or equal to .14 large. For Cramer's *V*, effect sizes range from 0 to 1 and indicate the strength of association between two variables. All other analyses were conducted using a combination of functions from base R and the furniture package (Barrett & Brignone, 2017). Figures were created using the ggplot2 (Wickham, 2016) and papaja (Aust & Barth, 2020) packages.

## 3 RESULTS

All participants (*N* = 104) self‐identified as White, and one participant as Hispanic/Latino. The four demographic groups (young men, young women, older men, older women) were relatively similar across all sample‐descriptive measures (e.g., years of education, physical health, mental health; see Table S1).

### 3.1 *OXTR* genotypes and alcohol use

Of the 56 participants who were genotyped, three participants (5.40%) had the A/A, 24 (42.90%) the G/A, and 29 (51.79%) the G/G genotype for *OXTR* rs53576. Thus, there were 27 participants in the combined X/A group (48.21%). Reporting regular alcohol consumption was associated with *OXTR* rs53576 genotype but quantities consumed were not. Specifically, A‐allele carriers less frequently reported regular use (33.00%) than did G/G homozygotes (62.00%); this difference was significant (*p* = .04, *V* = .26). Among those individuals who regularly consumed alcohol, there was no significant difference in self‐reported alcohol consumption between A‐allele carriers and G/G homozygotes (*W *= 86, *p* = .82, η^2 ^= .017).

### 3.2 Plasma OT and alcohol use

**FIGURE 1:**
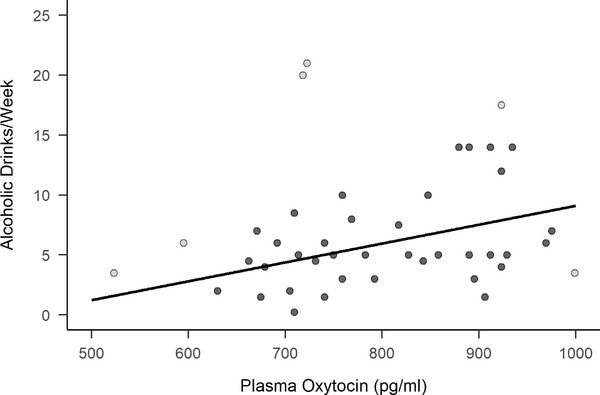
Number of alcoholic drinks consumed per week as a function of plasma oxytocin (OT) levels (in picogram by milliliters). Light gray data points indicate that the observation was considered highly influential and subsequently removed in post‐diagnostic regression analysis

Peripheral OT levels were positively associated with quantity of alcohol consumed among those who reported regular use of alcohol (see Figure 1). However, this association was only significant in the postdiagnostic model (*p* = .01). Table 1 contains test statistics and estimates for the overall model and coefficients for both the initial and postdiagnostic models; and Figure 1 includes the line of best fit from the postdiagnostic model.

**TABLE 1:** Test statistics and parameter estimates for bivariate regressions of plasma oxytocin (OT) concentrations and methylation of two oxytocin receptor (OXTR) cytosine–Guanine dinucleotide (CpG) sites predicting weekly alcoholic drinks

Model	Term	*N*	*F*	df	*p*	B (SE)	*t*	*p*
All observations		43	1.91	1, 41	.17			
	Intercept					−0.53 (5.39)	−0.10	.92
	Plasma OT					0.01 (0.01)	1.38	.17
Postdiagnostics		37	7.30	1, 35	.01			
	Intercept					−6.64 (4.73)	−1.41	.17
	Plasma OT					0.02 (0.01)	2.70	.01
								
All observations		27	6.26	1,25	.02			
	Intercept					13.50 (2.54)	5.32	<.001
	*OXTR* methylation (−860)					−0.26 (0.10)	−2.50	.02
Postdiagnostics		24	6.42	1, 22	.02			
	Intercept					13.07 (2.62)	4.99	<.001
	*OXTR* methylation (−860)					−0.26 (0.10)	−2.53	.02
								
All observations		27	0.95	1, 25	.34			
	Intercept					12.49 (5.19)	2.41	.02
	*OXTR* methylation (−924)					−0.08 (0.08)	−0.97	.34
Postdiagnostics		23	7.25	1, 21	.01			
	Intercept					26.08 (7.15)	3.65	.002
	*OXTR* methylation (−924)					−0.30 (0.11)	−2.69	.01

### 3.3 *OXTR* DNA methylation and alcohol use

**FIGURE 2:**
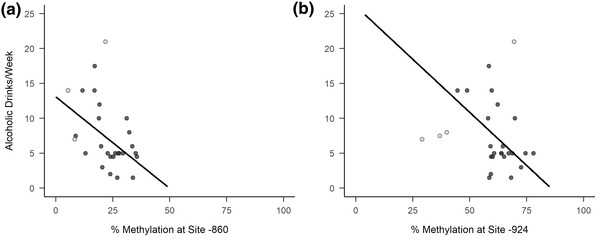
Number of alcoholic drinks consumed per week as a function of degree of oxytocin receptor gene (*OXTR*) methylation of two different promoter sites: −860 (a) and −924 (b). Light gray data points indicate that the observation was considered highly influential and subsequently removed in post‐diagnostic regression analyses

Levels of *OXTR* methylation were related to quantities of alcohol consumed. Methylation at *OXTR* CpG site −860 was associated with alcoholic drinks consumed per week both in initial and postdiagnostic models (*p*s = .02; see Table 1 for test statistics and coefficients). A scatterplot of alcoholic drinks per week as a function of *OXTR* methylation at the −860 site, including the line of best fit from the postdiagnostic model, is shown in Figure 2(a). Lower levels of methylation were associated with greater amounts of alcohol consumed per week. This association was comparable with *OXTR* methylation levels at site −924 and alcohol consumed per week (see Figure 2(b)), although for this CpG site it was only significant in the postdiagnostic model (*p* = .01; test statistics and coefficients also provided in Table 1).

No inferential tests involving *OXTR* CpG site −934 were significant and thus are not further discussed for brevity. For those interested, this measure is included in the corresponding data file on the Open Science Framework (Rung et al., 2020).

## 4 DISCUSSION

The present research encompasses a broad evaluation of the role of individual differences in the OT system in low to moderate alcohol use among younger and older adult humans. We found that greater self‐reported alcohol use was associated with higher levels of peripheral (plasma) OT and less *OXTR* methylation (at sites −860 and −924); and that regular alcohol use was more common among adults with the G/G genotype of *OXTR* rs53576.

Ongoing discussions in the field suggest that lower levels of OT activity may be a predisposing factor for substance use (Buisman‐Pijlman et al., 2014). Prior research documents significant associations between lower levels of plasma OT, greater *OXTR* methylation (Maud et al., 2018), and polymorphisms of *OXTR* (i.e., A‐allele carriers) to poorer rearing conditions (Branchi et al., 2013; Henriques et al., 2014) and poorer or impaired socioemotional functioning and development (Bakermans‐Kranenburg & van Ijzendoorn, 2008; Maud et al., 2018; Rodrigues et al., 2009). Based on these findings, we anticipated that alcohol use would be associated with lower plasma OT, greater *OXTR* methylation, and A‐allele carriers of the *OXTR* gene. However, our findings were largely contrary to these expectations. In our sample of individuals who drank at generally low to moderate levels, we found that lower peripheral OT and *OXTR* genotypes previously associated with more frequent alcohol use and increased likelihood of developing alcohol use disorder (AUD; Vaht et al., 2016) were associated with *less* weekly alcohol consumption and a *lower* likelihood of regular alcohol use. *OXTR* DNA methylation findings were in directional agreement with the aforementioned results: among regular consumers of alcohol, drinking *less* per week was associated with greater levels of *OXTR* methylation at both the −860 and −924 CpG sites (i.e., less *OXTR* expression).

These findings in our nonclinical, age‐heterogeneous sample importantly qualify and advance those previously documented. Several factors may explain why the direction of associations reported here differed from those in the (still rather limited) existing literature. We next outline several potential explanations with the intent to provide an impetus and further qualification for research on this topic.

First, the association between alcohol use and plasma OT among those individuals who regularly consumed alcohol but generally did so under the limits of harmful use may be more reflective of the effects of acute alcohol use on OT activity than the converse. In fact, preclinical evidence largely indicates that chronic exposure to a variety of drugs, including alcohol, reduces OT production and signaling (Lee et al., 2016; Light et al., 2004; Peris et al., 2020). Reduced OT production is often accompanied by increased *OXTR* expression, which suggests a compensatory mechanism for production deficits (see Bowen & Neumann, 2017). Though human research specific to alcohol use and OT activity is scarce, one study has shown that individuals with AUD had elevated plasma OT relative to controls, from 1 to 28 days of abstinence (Marchesi et al., 1997). These effects of alcohol on OT may be a downstream effect of changes in estrogen, as alcohol increases estrogen in both men and (pre)menopausal women (Emanuele & Emanuele, 1998; Ginsburg, 1999; Hansen et al., 2012), and estrogens are well‐known for their role in upregulating OT release and *OXTR* transcription (Dhakar et al., 2013). Other research has shown reduced OT immunoreactivity in postmortem hypothalamic tissue (Sivukhina et al., 2006) and increased *OXTR* mRNA expression levels in the prefrontal cortex among individuals with AUD (Lee et al., 2017), both of which are putatively indicative of lower OT activity with chronic alcohol use. Thus, the generally opposite associations found in the present study, when compared to findings from studies on individuals with heavier levels of alcohol use, may represent acute effects that over more prolonged and intense durations would eventually lead to downregulation and desensitization of the OT system.

Given the above findings, it is also plausible that the higher levels of plasma OT in our sample are indicative of a rebound‐like (i.e., withdrawal) effect; whereas the lower plasma OT levels, which were among those individuals with lower weekly alcohol consumption, may better represent baseline circulating levels. Unfortunately, we did not ask participants about the time since last consuming an alcoholic beverage. However, the timeframe of the questions asked (typical weekly use) implies that last use was likely in the past 7 days. That the effects of alcohol on plasma OT are evident for a prolonged duration support this interpretation (Marchesi et al., 1997), although subsequent research has shown that not only is OT impacted for quite some time following abstinence, but it does so dynamically and differentially as a function of dependence (Hansson et al., 2018). As such, it is imperative that future studies obtain time of last drink to better evaluate this speculation.

If indeed our measures of plasma OT are reflective of the impact of acute exposure or a withdrawal‐like effect in the heavier drinkers, then the question of whether endogenous OT is predictive of alcohol use more generally, and harmful use specifically, remains. Such questions are more amenable to longitudinal designs, repeated measures before and after initiation of alcohol use, and comparative studies among acute and chronic users. It is important to keep in mind that most participants in the present sample often consumed alcohol within low‐risk weekly limits (≤7 drinks per week for women; ≤14 drinks per week for men; NIAAA, 2017), with only five (11.6%) females and two (4.7%) males exceeding them. The speculation that plasma OT levels may be indicative of withdrawal‐like effects among heavier drinkers in particular is grounded in large part on findings from individuals diagnosed with AUD who consumed significantly greater quantities of alcohol: about 13.5 drinks per day (Marchesi et al., 1997), in contrast to approximately 1 drink per day in the present sample.

Second, findings pertaining to the effects of OT on behavior (Bartz et al., 2011), as well as in the relations between endogenous OT and constructs/conditions of interest (e.g., levels of plasma OT and depression; Cochran et al., 2013), have been somewhat variable in significance and/or direction across studies. This heterogeneity may be viewed as a sign of context dependency through which environmental and person‐level moderators of OT are revealed (Bartz et al., 2011; Carter et al., 2020; Olff et al., 2013). Such considerations also apply to measures of *OXTR* methylation: some studies have proposed that greater methylation may be uniformly associated with decrements in various cognitive and affective processes, whereas the relations may be the opposite (or more complex) across diagnostic phenotypes (Maud et al., 2018). Conducting research in the future that targets, for example, adults who consume alcohol with versus without a diagnosis of mood and/or anxiety disorder could address these questions. Including questions specific to mental health diagnoses would facilitate conducting subgroup analyses in nontargeted samples. In the present study, participants were not directly asked about such conditions, and few (*n* = 3) reported having any when asked about having any major health conditions. We ensured that these three participants’ data did not bias the present findings,^1^ but the low frequency of such conditions among our participants precludes meaningful evaluation of the effects such conditions may have (i.e., via subgroup analysis or inclusion of mental health condition as a covariate).

Third, heterogeneity in study findings involving individual differences in the OT system may also be due to differing methods of measuring OT markers. Across studies, methylation is often evaluated at a range of different cytosine–guanine dinucleotide (CpG) sites and using different cells for conducting assays (e.g., buccal vs. blood; see Kraaijenvanger et al., 2019, for discussion). Different cell sources and methylation from varying CpG sites may serve as better proxies of *OXTR* methylation in different neural regions and substrates (e.g., Gregory et al., 2009).

A final, potential explanation of some of the present findings is that the measures of peripheral OT and *OXTR* DNA methylation are interdependent. For example, if peripheral levels of OT are increased as a means of compensating for the effects of greater methylation, then this may account for the parallel, significant correlations between these measures and alcohol use. However, in our study peripheral OT was not significantly correlated with the methylation measures at any CpG site (see Table S2). As such, this explanation seems unlikely to account for the present findings.

Together, these considerations give rise to numerous future directions for furthering our understanding of the role of the OT system in alcohol use more generally and its viability as a target for substance use disorders, including among generally healthy men and women of different ages. Perhaps most importantly from a prevention standpoint, more longitudinal research is needed in which both substance use and measures of the OT system are tracked across time. For example, to date, there is still no research that has evaluated dynamic markers of OT (i.e., endogenous OT and *OXTR* methylation) and substance use initiation. These types of studies will help parse apart the role of baseline levels of OT functioning, the effects of substance use on measures thereof, and other related processes such as social support (e.g., Kogan et al., 2018).

Some limitations of the present research include the lack of a standard measure of alcohol use and an explicit assessment of current or prior AUD. The present research questions were inspired by emerging research and reviews on the relevance of OT to substance use but were secondary in nature and formed after data collection was completed. As such, we used measures that were available and standardized them to the extent possible. Future research that goes beyond these initial steps should employ measures of alcohol use that include the definition of a standard drink and involve a longer as well as a finer‐grained period of assessment (e.g., the Timeline Followback [TLFB] covering the past 30 days; Sobell & Sobell, 1992). In addition to allowing a more precise calculation of drinks per week (or day) and time since last drink, assessments such as the TLFB allow calculation of the number (or proportion) of moderation drinking days (≤1 standard drink per day for women and ≤2 standard drinks per day for men) and binge drinking days (>3 or >4 drinks on a single day for women and men, respectively; NIAAA, 2017). In the absence of targeted screening for AUD, the TLFB can be used to quantify risk for AUD based on drinking practices. Although, incorporation of screening tools for use disorder or measures of alcohol consequences would also be beneficial moving forward (e.g., the Alcohol Use Disorders Identification Test; Saunders et al., 1993). While the measurement of alcohol consumption for the present study was not as extensive as these standard measures, the observed consistency in associations across the measures of peripheral and (epi)genetic OT markers highlights implication of the OT system in alcohol use and the need for further research.

Nicotine use was rare in this sample (five participants, 4.8% of the sample) and had little impact on the present results.^2^ However, cotinine levels have shown positive associations with OT among smokers with AUD, but no significant associations between self‐reported cigarettes per day and OT (Haass‐Koffler et al., 2021). There is a paucity of studies to date investigating the co‐use of alcohol and tobacco/nicotine and associations with individual differences in the OT system (Haass‐Koffler et al., 2021). Exogenous OT administration has been shown to reduce craving for cigarettes and cigarette smoking compared to placebo within a laboratory paradigm (Van Hedger et al., 2019). More research in this area would be of value, as smoking is associated with greater likelihood of AUD, greater binge drinking, and more alcohol‐related problems (McKee & Weinberger, 2013); and OT has been suggested as a potential therapeutic for both alcohol and nicotine use disorders (Mitchell et al., 2016; Tunstall et al., 2019; Van Hedger et al., 2019).

Another limitation of the present study was the disproportionately larger representation of older adults in the analyses pertaining to epigenetic and genetic variability in *OXTR* (approximately 60% older adults). This was a byproduct of implementing the (epi)genotyping assays sometime after data collection had commenced. While this unevenness could have introduced bias in the results of analyses involving *OXTR* genotypes and methylation, the proportion of older adults was still relatively close to 50%, and other variables (e.g., plasma OT, physical and mental health) were similar across genotyped and non‐genotyped participants. Between this and the lack of other significant differences across age/sex groupings (e.g., Tables S1 and S4), the differential proportional composition across analysis subsets seems unlikely to have played a major role in the present findings. However, such questions could be better addressed in future research with larger *N*s to explicitly examine age as a potential moderator.

Another priority for future studies is to ensure adequate representation. While our sample was age diverse, it was homogeneous in race and ethnicity. Because of the relatively small sample size, study enrollment was restricted to those identifying as white (i.e., the majority in the region of recruitment) because cultural and social factors have been shown to moderate relations between *OXTR* genotypes and outcomes of interest. For instance, ethnicity moderates the relation between *OXTR* genotypes and engagement in emotion suppression (Kim et al., 2011); and allelic associations with autism spectrum disorder generalize across Chinese and Japanese samples but not white (Liu et al., 2010). Thus, in this study, it was important to control for confounding factors that could otherwise mask effects. Future research should plan for powered sample sizes to allow the evaluation of moderations by facets of diversity or by recruiting larger diverse samples. Doing so will be important for understanding nuances of the role that the OT system plays in substance use and substance use disorder across a wide range of individuals.

Further extending the approach taken in our research, future studies should employ diverse measures pertaining to OT activity and (epi)genetic variation. Given that *OXTR* genotypes are thought to impact OT signaling, neither endogenous OT nor OT genotypes alone may be robust predictors of substance use. For example, ongoing research suggests that the effects of or associations between *OXTR* methylation and phenotypes are dependent on *OXTR* genotypes (Kraaijenvanger et al., 2019). While all these measures were obtained in the present research, we did not conduct analyses involving genotype/epigenetic measures by endogenous OT interactions due to the modest sample size for such moderator analyses. In addition to the cross‐sectional and longitudinal evidence previously discussed, there are a variety of experimental applications demonstrating protective effects of exogenous OT administration (Lee et al., 2016; McGregor & Bowen, 2012). For instance, OT administration reduces self‐administration of a variety of substances in non‐humans, including alcohol (Bowen et al., 2011), methamphetamine (Hicks et al., 2016), and heroin (Kovács et al., 1985). OT administration also reduces conditioned place preference (i.e., reduces the rewarding properties of substances) for methamphetamine (Carson et al., 2010) and oxycodone (Fan et al., 2019) in rats. These findings complement the notion that greater OT activity, modeled by exogenous administration, reduces propensity to engage in substance use.

### 4.1 Conclusions

A growing body of preclinical and basic human research suggests that lower OT activity may increase susceptibility to harmful substance use and/or the development of substance use disorders. Most research on this topic in humans speaks to the shared antecedents of substance use and their relations to measures of OT activity and genetic variation (e.g., research on the effects of early life adversity). The present research found significant relations between measures of OT activity and genetic variation and alcohol use, thereby supporting the relevance of the OT system in alcohol use. Given the low levels of alcohol use in the present sample relative to those seen in AUD, the findings may best reflect the role of OT in alcohol use when under the control of its rewarding properties and/or the effects of alcohol on OT activity itself. Additional research is needed with larger samples, standard measures of alcohol use, and over longer durations to better understand the complexities of the role of OT function in the use of alcohol and other substances. Collectively, this knowledge will help researchers better characterize the neurobiological determinants of substance use and potentially important points of and markers for prevention.

## CONFLICT OF INTEREST

The authors have no conflicts of interest to declare.

### PEER REVIEW

The peer review history for this article is available at https://publons.com/publon/10.1002/brb3.2425

## Supporting information

Supporting information

## Data Availability

The data that support the findings of this study are openly available on the Open Science Framework, which can be accessed via DOI https://doi.org/10.17605/OSF.IO/JWN6B. Full reference information for the data and analysis code is provided in the reference section (Rung et al., 2020).

## Footnotes

1 The mental health conditions reported by the three participants were: depression, bipolar disorder, and anxiety disorder. None of these individuals reported regularly consuming alcohol, and as such their data were only included in the analysis of differences in proportions of regular alcohol consumed by *OXTR* genotypes (G/G vs. A‐allele carriers). The effect, while no longer statistically significant, was similar in magnitude to that with all participants included (*V* = .25 without vs. *V* = .26 with). The lack of significance is likely due to the reduced analytic *N*. The respective code is included in the analysis file on the Open Science Framework that identifies these three participants with mental health conditions, which can also be used to filter these individuals out of the analyses.

2 We conducted all primary analyses with and without the five participants who reported current nicotine use (3 cigarette smokers and 2 cigar smokers). All conclusions pertaining to statistical significance were the same, with the exception of one test which revealed a slightly reduced effect size when removing these participants. The affected test was the one comparing differences in proportions of regular alcohol consumers by *OXTR* genotypes (G/G vs. A‐allele carriers), which was *V* = .24 without nicotine users and *V* = .26 with all observations included. The respective data and code can be found on the Open Science Framework (Rung et al., 2020).
