# Supplementary material for: Associations between alcohol use and peripheral, genetic, and epigenetic markers of oxytocin in a general sample of young and older adults
Source: Brain Behav. 2022 Feb 11;12(3):e2425. doi: 10.1002/brb3.2425 (PMC8933764; doi:10.1002/brb3.2425)
Supplement: Supplementary file 1 — Supporting information [file BRB3-12-e2425-s001.docx]

Table S1. Descriptive statistics, comparisons, and effect sizes (η^2^/Cramer’s *V)* thereof of demographic variables, participant characteristics, and classifications and weekly quantities of alcohol consumed across the four age/sex groupings.

|  | Younger Adults | | | | Older Adults | | | |  |  |
| --- | --- | --- | --- | --- | --- | --- | --- | --- | --- | --- |
|  | Female (*n* = 24) | | Male (*n* = 27) | | Female (*n =* 30) | | Male (*n =* 23) | | Effect Size | *p* |
| Variable | *n* (%) | Median (Q1-Q3) | *n* (%) | Median (Q1-Q3) | *n* (%) | Median (Q1-Q3) | *n* (%) | Median (Q1-Q3) |  |  |
| Age (years) |  | 22 (21-25) |  | 21 (20-24) |  | 70 (67-74) |  | 74 (68-76) | .75 | <.001 |
| Ethnicity |  |  |  |  |  |  |  |  | .18 | .32 |
| Not Hispanic/  Latinx | 24 (100%) |  | 26 (96.3%) |  | 30 (100%) |  | 22 (95.7%) |  |  |  |
| Hispanic/  Latinx | 0 (0%) |  | 1 (3.7%) |  | 0 (0%) |  | 0 (0%) |  |  |  |
| N/A | 0 (0%) |  | 0 (0%) |  | 0 (0%) |  | 1 (4.3%) |  |  |  |
| Years of Education |  | 16 (14-17) |  | 15 (14-16) |  | 16 (14-18) |  | 17 (14-18) | .01 | .26 |
| Physical Health |  | 9 (7-9) |  | 9 (8-9) |  | 9 (8-9) |  | 9 (8-9) | .006 | .50 |
| Mental Health |  | 8 (8-9) |  | 9 (8-10) |  | 9 (8-10) |  | 9 (9-9) | .015 | .22 |
| Regular Alcohol Use |  |  |  |  |  |  |  |  | .24 | .13 |
| No | 17 (70.8%) |  | 11 (40.7%) |  | 20 (66.7%) |  | 13 (56.5%) |  |  |  |
| Yes | 7 (29.2%) |  | 16 (59.3%) |  | 10 (33.3%) |  | 10 (43.5%) |  |  |  |
| Alcoholic Drinks/Week |  | 5.0 (4.0-6.0) |  | 5 (4.0-9.4) |  | 7.5 (5.0-13.0) |  | 4.2 (1.9-7.1) | .013 | 0.23 |

Note: Medians and first and third quartiles are presented due to violations of normality within groupings (*p* < .05; based on Shapiro-Wilks tests). Kruskal Wallis tests involving continuous variables are conducted treating the age/sex grouping as a categorical variable with four levels representing each grouping. The effect size provided is either η^2^ (calculated using the Kruskal-Wallis test statistic) or Cramer’s *V* for continuous and frequency data, respectively. Age, years of education, and physical and mental health ratings are rounded to the nearest whole number. All measures were based on self-report.

Table S2. Pearson correlations between levels of plasma oxytocin (OT) and *OXTR* DNA methylation at three CpG sites (-860, -924, -924).

| Measure | 1. | 2. | 3. | 4. |
| --- | --- | --- | --- | --- |
| 1. Plasma OT | 1.00 | - |  |  |
| 2. Site -860 | -0.20 | 1.00 |  |  |
| 3. Site -924 | 0.12 | 0.46* | 1.00 |  |
| 4. Site -934 | 0.13 | 0.50* | 0.72* | 1.00 |

**p* < .001

Table S3. Frequencies (percentages), comparisons, and effect sizes (Cramer’s *V*) thereof of the *OXTR* gene variant rs53576 (original and recoded) across the four age/sex groupings.

|  | Younger Adults | | Older Adults | | *V* | *p* |
| --- | --- | --- | --- | --- | --- | --- |
| *OXTR* Genotype | Female (*n* = 24) | Male (*n* = 27) | Female (*n* = 30) | Male (*n* = 23) |  |  |
| Original |  |  |  |  | .14 | .92 |
| A/A | 0 (0%) | 1 (3.7%) | 1 (3.3%) | 1 (4.3%) |  |  |
| G/A | 4 (16.7%) | 4 (14.8%) | 8 (26.7%) | 8 (34.8%) |  |  |
| G/G | 5 (20.8%) | 8 (29.6%) | 10 (33.3%) | 6 (26.1%) |  |  |
| n/a | 15 (62.5%) | 14 (51.9%) | 11 (36.7%) | 8 (34.8%) |  |  |
| Recoded |  |  |  |  | .16 | .73 |
| G/G | 5 (20.8%) | 8 (29.6%) | 10 (33.3%) | 6 (26.1%) |  |  |
| A/X | 4 (16.7%) | 5 (18.5%) | 9 (30%) | 9 (39.1%) |  |  |
| n/a | 15 (62.5%) | 14 (51.9%) | 11 (36.7%) | 8 (34.8%) |  |  |

*Note:* Significance values are from Fisher’s Exact tests comparing the frequencies of the different genotypes (and those who did not have genotyping performed) across all four age/sex groups. A/X indicates that a participant was an A-allele carrier (hetero- or homozygous); n/a indicates genotyping was not conducted.

Table S4. Descriptive statistics (means/standard deviations; or medians and 1^st^ and 3^rd^ quartiles), comparisons (ANOVAs or equivalent), and effect sizes thereof of degree of *OXTR* methylation at two different cytosine–guanine dinucleotide (CpG) sites across the four age/sex groupings, for all participants and among those reporting regular alcohol use.

|  |  | Younger Adults | | Older Adults | |  |  |
| --- | --- | --- | --- | --- | --- | --- | --- |
| Subset | *OXTR* CpG  Site | Female | Male | Female | Male | η^2^ | *p* |
| All |  | *n* = 9 | *n* = 13 | *n* = 19 | *n* = 15 |  |  |
|  | -860 | 27.4 (5.8) | 22.2 (3.4) | 24.8 (8.8) | 24.0 (10.7) | .043 | .51 |
|  | -924 | 64.0 (62.1-64.6) | 60.1 (59.1-68.3) | 64.7 (57.5-69.6) | 64.5 (58.3-70.7) | .026 | .94 |
|  |  |  |  |  |  |  |  |
| Regular Alcohol Users |  | *n* = 4 | *n* = 8 | *n* = 8 | *n* = 7 |  |  |
|  | -860 | 30.1 (5.4) | 21.6 (4.0) | 23.7 (11.2) | 19.1 (8.9) | .175 | .21 |
|  | -924 | 64.2 (62.8-64.7) | 59.8 (59.3-61.1) | 65.5 (43.4-68.8) | 58.6 (53.4-70.3) | .068 | .95 |

*Note*: Medians (and first and third quartiles) are reported for *OXTR* methylation measures at site -924 because this variable evidenced non-normality in at least one of the age/sex groupings, in all subsets (Shapiro-Wilks test, *p* < .05). In such cases, reported *p*-values are from the non-parametric equivalent to ANOVA (Kruskal-Wallis tests). Tests are conducted treating the age/sex grouping as a categorical variable with four levels to represent each grouping.
